# Low-dose urokinase thrombolysis combined with papaverine for central retinal artery occlusion: a preliminary exploration of efficacy and safety

**DOI:** 10.3389/fneur.2025.1760868

**Published:** 2026-01-12

**Authors:** Hongyan Sun, Yang Geng, Wenchao Qiu, Jiahao Zhu, Mengxin Jiang, Zengqiang Xu, Wenming Wang, Yingjiang Xu, Jianwei Gao

**Affiliations:** 1 Rongcheng Shidao People's Hospital, Rongcheng, Shandong, China; 2 Binzhou Medical University Hospital, Binzhou, Shandong, China; 3 School of Medical Imaging (Binzhou Medical University), Binzhou, Shandong, China

**Keywords:** central retinal artery occlusion, ophthalmic artery thrombolysis, papaverine, safety, urokinase, visual recovery

## Abstract

**Objective:**

To investigate the efficacy and safety of low-dose urokinase combined with papaverin administered via superselective ophthalmic artery thrombolysis in patients with non-arteritic central retinal artery occlusion (CRAO).

**Methods:**

A retrospective analysis was conducted on the clinical data of 20 CRAO patients who underwent ophthalmic artery thrombolysis at our hospital between December 2023 and July 2025. All procedures were performed under local anesthesia via femoral artery puncture and catheterization. A sequential injection of 400,000 IU urokinase followed by 30 mg papaverine was administered into the ophthalmic artery. Best-corrected visual acuity (BCVA, expressed in logMAR) was assessed pre- and post-operatively, and adverse events were recorded.

**Results:**

The median time from symptom onset to treatment was 7.25 h (IQR: 2.00–20.50). The mean preoperative BCVA in the affected eye was 2.38 ± 0.93 logMAR, which significantly improved to 0.33 ± 0.26 logMAR postoperatively (*p* < 0.001). The median improvement in BCVA was 2.54 logMAR units (IQR: 0.84–2.78). Although a statistically significant difference persisted between the postoperative visual acuity of the affected eye and that of the contralateral healthy eye (0.28 ± 0.26 logMAR) (*p* = 0.002), the median difference was only 0.075 logMAR, suggesting limited clinical significance. No serious adverse events, such as hemorrhage or cerebral infarction, occurred in any patient.

**Conclusion:**

Low-dose urokinase combined with papaverine via ophthalmic artery thrombolysis significantly improved visual acuity with a favorable safety profile in carefully selected CRAO patients, demonstrating potential for clinical application.

## Introduction

Central retinal artery occlusion (CRAO) is an ocular emergency characterized by abrupt monocular visual loss or decline due to interrupted blood flow in the central retinal artery (CRA), typically resulting from thromboembolism or vasospasm. CRAO significantly increases the risk of acute ischemic vascular events (e.g., stroke, acute myocardial infarction), mortality, and ocular neovascularization (ONV) (1, 2). Recognized as a form of acute ischemic stroke, the diagnosis, management, and prognosis of CRAO have improved in recent years, paralleling advancements in fundus and cerebral imaging techniques, growing acceptance of intravenous and intra-arterial thrombolysis, and refinements in acute ischemic stroke treatment protocols (3). The incidence of CRAO varies geographically and by demographic factors, with a global annual incidence estimated at 1–2 per 100,000. It predominantly affects middle-aged and elderly individuals, with a higher prevalence in males, and its incidence increases with age (3).

Current conservative management strategies for non-arteritic CRAO include anterior chamber paracentesis, pharmacological intraocular pressure reduction, and vasodilators. However, previous studies indicate limited efficacy of conservative treatments in promoting visual recovery, with reported success rates below 15% or even lower (3, 4). Intra-arterial thrombolysis (IAT) utilizes a microcatheter to deliver fibrinolytic agents directly into the ophthalmic artery (5), enabling targeted action on the thrombus within the CRA. This approach aims to rapidly dissolve the occlusion, restore blood flow, and mitigate ischemic damage (3, 6–9). This study aimed to evaluate the feasibility, efficacy, and safety of a specific low-dose regimen administered via superselective ophthalmic artery thrombolysis in patients with non-arteritic CRAO.

## Methods and materials

This was a retrospective, single-arm case series. As it was not a randomized controlled trial, the CONSORT guideline was not directly applicable. However, we have reported the study in accordance with the STROBE (Strengthening the Reporting of Observational Studies in Epidemiology) statement for observational studies to ensure comprehensive reporting.

### Study population

Inclusion criteria were: (1) clinical diagnosis of non-arteritic CRAO; and (2) treatment with superselective ophthalmic artery thrombolysis.

Exclusion criteria, based on comprehensive evaluation of detailed patient history, clinical presentation, fundus examination, optical coherence tomography (OCT), and ancillary tests, included: coagulation disorders, recent stroke, severe arterial hypertension, hemorrhagic diathesis, head trauma, recent gastrointestinal bleeding, proliferative diabetic retinopathy, hypertensive retinopathy, and retinal arteritis.

This retrospective case series study included 20 CRAO patients admitted to Rongcheng Shidao People’s Hospital, between December 2023 and July 2025, who met the inclusion and exclusion criteria. All procedures in this study were conducted and postoperatively managed at Shidao Hospital. The study protocol was reviewed and approved by the Ethics Committee of Shidao Hospital, Rongcheng (Approval No. 2025KYLL03), and all procedures adhered to the ethical principles outlined in the Declaration of Helsinki. Prior to surgery, all patients were thoroughly informed of the potential benefits and risks of the procedure and provided written informed consent.

### Procedure

All procedures were performed under local anesthesia. The femoral artery was punctured using the modified Seldinger technique, and a 5F vascular sheath was introduced. A 5F guiding catheter was navigated sequentially through the common femoral, iliac, and aortic arteries into the common carotid and subsequently the internal carotid artery (ICA). Cerebral angiography via the ICA was performed to confirm ophthalmic artery patency and identify its origin. A microguidewire and microcatheter were then used to superselect the ophthalmic artery. Follow-up angiography confirmed the occlusion site within the ophthalmic artery or its branches. Subsequently, 400,000 IU of urokinase followed by 30 mg of papaverine were slowly injected through the microcatheter. To minimize the risk of vasospasm and ischemia, the microcatheter dwell time within the ophthalmic artery was kept under 5 min. Angiography was repeated 30 min post-injection to assess reperfusion.

### Outcome measures

All patients underwent OCT angiography (OCTA) and ocular vascular ultrasound examinations pre- and post-operatively. Preoperative visual acuity was recorded using decimal notation and converted to the logarithm of the minimum angle of resolution (logMAR) for statistical analysis. Postoperative visual acuity was rechecked within 1–2 days.

### Statistical analysis

Data were analyzed using SPSS software (version 27). Normality of continuous variables was assessed using the Kolmogorov–Smirnov test. Descriptive statistics are presented as mean ± standard deviation (SD) for normally distributed continuous variables (e.g., age), and as median (interquartile range, IQR) for non-normally distributed continuous variables (e.g., onset-to-treatment time, logMAR visual acuity). Categorical variables are expressed as frequency (percentage). For group comparisons, the Wilcoxon signed-rank test was used to evaluate changes in visual acuity before and after treatment. Comparisons between the affected eye and the contralateral healthy eye were performed using the Mann–Whitney U test or Wilcoxon signed-rank test, as appropriate based on data pairing. The relationship between onset-to-treatment time and the degree of visual improvement was examined using Spearman’s rank correlation analysis. A two-sided *p* < 0.05 was considered statistically significant.

## Results

### Baseline characteristics and visual outcomes

A total of 20 patients with CRAO were included in this study. Demographic and baseline clinical characteristics are summarized in Table 1. The median duration from symptom onset to the initiation of thrombolytic therapy was 7.25 h (interquartile range [IQR]: 2.00–20.50).

**Table 1:** Patient demographics and baseline characteristics.

Characteristic	Statistic
Demographics	
Age (years), Mean ± SD	63.15 ± 11.60
Gender, *n* (%)	
Male	13 (65%)
Female	7 (35%)
Ocular characteristics	
Affected Eye, *n* (%)	
Left	9 (45%)
Right	11 (55%)
Onset to Treatment Time (h), Median (IQR)	7.25 (2.00, 20.50)
Preop Affected Eye logMAR BCVA, Median (IQR)	3.00 (1.25, 3.00)
Postop Affected Eye logMAR BCVA, Median (IQR)	0.22 (0.11, 0.49)
Contralateral Eye logMAR BCVA, Median (IQR)	0.19 (0.10, 0.28)
Comorbidities, *n* (%)	
Hypertension	8 (40%)
Diabetes Mellitus	6 (30%)
Coronary Artery Disease	3 (15%)
The *p*-value for preoperative vs. postoperative BCVA	*p* < 0.001

### Efficacy of thrombolysis

**Figure 1:**
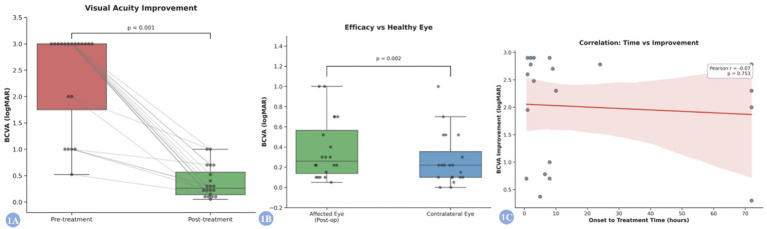
Visual outcomes and prognostic analysis following intra-arterial thrombolysis. **(A)** Paired boxplot demonstrating the significant improvement in best corrected visual acuity (BCVA) before and after treatment. Individual gray lines represent the trajectory of each patient, showing a consistent downward trend (lower logMAR indicates better vision). The difference was statistically significant (Wilcoxon signed-rank test, *p* < 0.001). **(B)** Boxplot comparing the postoperative BCVA of the affected eyes with the baseline BCVA of the contralateral healthy eyes. Although the treated eyes showed marked improvement, a statistically significant difference persisted compared to the healthy fellow eyes (*p* = 0.002). **(C)** Scatter plot with linear regression analysis illustrating the relationship between the time from symptom onset to treatment and the magnitude of visual improvement. A positive correlation was observed, indicating that shorter onset-to-treatment times tend to result in greater visual gains.

Following intra-arterial thrombolysis with low-dose urokinase and papaverine, visual function improved significantly. The mean Best Corrected Visual Acuity (BCVA) of the affected eyes improved from a baseline of 2.38 ± 0.93 logMAR to 0.33 ± 0.26 logMAR postoperatively. A Wilcoxon signed-rank test confirmed a statistically significant reduction in logMAR values (Figure 1A; *Z* = −3.927, *p* < 0.001). The median improvement in BCVA was 2.54 logMAR units (IQR: 0.84–2.78), indicating a substantial and clinically meaningful visual gain.

### Comparison with contralateral healthy eyes

Although visual acuity in the affected eyes improved markedly, it did not fully reach the baseline level of the contralateral healthy eyes (mean: 0.28 ± 0.26 logMAR). A paired comparison revealed a statistically significant difference between the postoperative affected eyes and the healthy fellow eyes (Figure 1B; median difference = 0.075 logMAR, IQR: 0.000–0.120; *Z* = 3.064, *p* = 0.002). However, given the small magnitude of this difference (approximately 1 line on the Snellen chart), its clinical relevance may be limited. Notably, complete functional recovery (defined as a visual acuity difference of 0 logMAR compared to the healthy eye) was achieved in 8 patients (40%), underscoring the potential of this regimen for full visual restoration.

### Prognostic factors and safety

**Figure 2:**
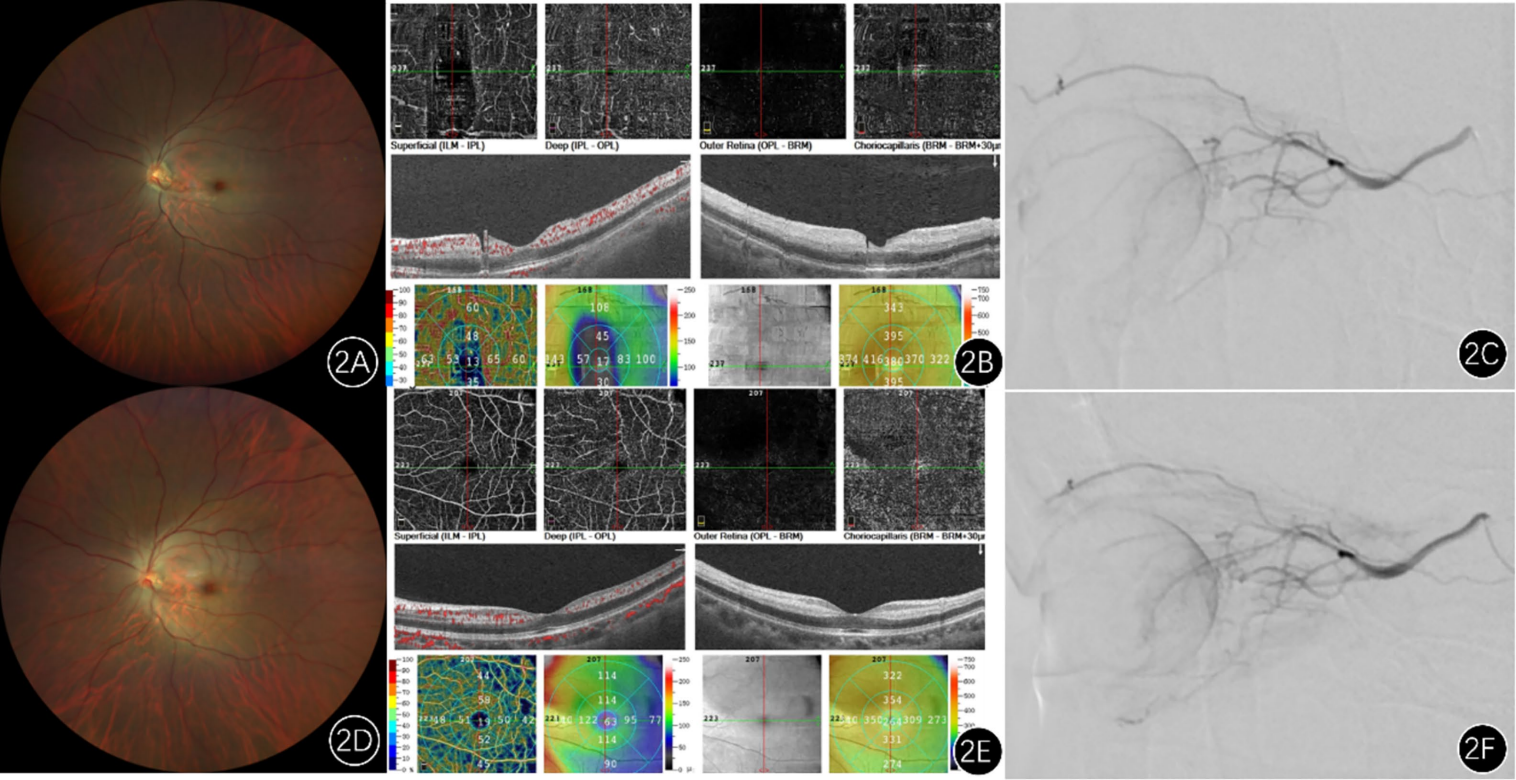
Imaging findings of a patient with left central retinal artery occlusion. **(A)** Pretreatment fundus photograph shows attenuated retinal arteries and veins (arteriovenous ratio approximately 2:3), with slightly darkened veins compared to normal, pallor of the posterior pole retina, and a classic cherry-red spot in the macular region. **(D)** Post-intra-arterial thrombolysis fundus photograph reveals dilation of the retinal vessels and increased blood flow compared to pretreatment. **(B)** Preoperative optical coherence tomography (OS): demonstrates thickening and increased reflectivity of the inner retinal layers, alongside reduced signal from the outer retinal layers. Optical coherence tomography angiography: shows decreased vessel density in the macular region. **(E)** Postoperative imaging shows improved macular vessel density compared to the preoperative state. **(C,F)** Represent angiography images before and after intra-arterial thrombolysis, respectively, revealing markedly increased blood flow following thrombolysis.

Further analysis demonstrated a correlation between treatment timing and visual outcomes (Figure 1C), suggesting that earlier intervention is associated with better visual recovery. Regarding safety, no treatment-related adverse events, such as hemorrhage, cerebral infarction, arterial dissection, or pseudoaneurysm, were observed. A representative case illustrating the imaging changes is shown in Figure 2.

## Discussion

### Feasibility of intra-arterial thrombolysis and pathophysiological basis

CRAO represents an acute ischemic stroke of the retina, sharing pathophysiological similarities with cerebral artery occlusion. Theoretically, early restoration of blood flow is crucial for salvaging ischemic retinal tissue. IAT, involving the direct administration of thrombolytic agents into the ophthalmic artery via a microcatheter, aims to achieve high local drug concentrations, maximize thrombus dissolution, and minimize systemic side effects. The core pathophysiological rationale hinges on restoring perfusion before irreversible retinal damage occurs (6, 8, 9). Seminal animal experiments by Hayreh et al. in elderly, atherosclerotic rhesus monkeys elucidated a critical time window for retinal survival: if CRAO duration is less than approximately 97 min, detectable retinal injury may be absent; whereas ischemia lasting beyond approximately 240 min (4 h) results in severe and irreversible retinal damage (10). This finding provides the fundamental theoretical basis for all reperfusion therapies, including IAT, and underscores the paramount importance of early intervention. By delivering drugs directly within this putative “time window,” IAT seeks to rapidly dissolve the thrombus and maximize rescue of jeopardized retinal neurons while reducing systemic exposure.

However, the role of IAT in CRAO management remains contentious. The EAGLE study (11), the only multicenter randomized controlled trial to date comparing IAT versus conservative treatment, found no significant difference in visual improvement between groups and reported a higher incidence of adverse events in the IAT group (37.1% vs. 4.3%), leading to the conclusion that IAT should not be recommended as standard care. Critically, the mean time to treatment in the EAGLE study was 13 h, substantially exceeding the 4-h ideal window suggested by animal studies. Thus, its results likely reflect the inefficacy of late intervention on already infarcted retina. Conversely, several retrospective studies support the efficacy of IAT when performed within a shorter time frame. For instance, Weber et al. (12) reported that 29.4% of patients in the IAT group achieved visual acuity better than 20/30. A Scientific Statement from the American Heart Association also notes that, despite limited evidence, IAT may be a consideration in selected early-presenting (especially within 4.5 h) CRAO cases at experienced centers, warranting further investigation (3). In our study, the median treatment time was 7.25 h, partially beyond the ideal window, yet significant visual improvement was still observed. This suggests that IAT retains clinical potential in carefully selected patients, particularly if treatment can be initiated earlier. This delay likely reflects real-world challenges in patient presentation and referral. The significant visual improvement observed even beyond this window suggests that the ‘point of no return’ for retinal ischemia in humans may exhibit individual variability, possibly influenced by collateral circulation (e.g., cilioretinal arteries) or incomplete occlusion. However, the positive correlation we observed between earlier treatment and better outcome (Figure 1C) reinforces the imperative for ultra-early intervention. This finding may, of course, also be attributable to our single-center, small-sample design, which carries inherent bias and stochastic uncertainty. Future studies should stratify outcomes by precise treatment timelines.

### Rationale for low-dose urokinase and safety considerations

Urokinase, a non-specific plasminogen activator, possesses potent thrombolytic activity, but its dosage correlates with bleeding risk (13). The 400,000 IU urokinase dose adopted at our center was derived from cumulative clinical experience rather than from formal dose-finding studies. This choice balances two considerations: on one hand, animal studies indicate extreme sensitivity of retinal neurons to ischemia, necessitating rapid reperfusion, which requires an effective thrombolytic agent; on the other hand, the fragility of the ophthalmic and intracranial vasculature means higher thrombolytic doses significantly increase hemorrhage risk, including potentially fatal intracranial bleeding. Therefore, the low-dose strategy aims to equilibrate thrombolytic efficacy and hemorrhagic risk, particularly suited for tissues with dense microvasculature like the retina.

One study suggested that low-dose urokinase can achieve vascular recanalization without significantly increasing bleeding events (13). The absence of hemorrhagic complications in our cohort may be attributed not only to controlled drug dosage but also potentially to the management within a feasible time window–delayed intervention beyond the critical period might involve compromised blood-retinal barrier integrity, potentially increasing hemorrhage risk even with thrombolytics while offering diminished recanalization benefit. Furthermore, urokinase’s short half-life and rapid metabolism following local administration may contribute to reduced systemic risk.

### Theoretical basis for combining papaverine and mechanism of synergistic action

This study combined urokinase with papaverine (30 mg) to alleviate vasospasm and improve microcirculatory flow. Papaverine, a non-specific vasodilator, inhibits phosphodiesterase, increases intracellular cAMP levels, and relaxes vascular smooth muscle. It is particularly relevant in CRAO cases with a vasospastic component (14). Vasodilation induced by papaverine may potentially enhance the penetration and efficacy of subsequently administered urokinase at the thrombus site. Moreover, in cases where the occlusion comprises cholesterol or calcific emboli less responsive to thrombolysis (15), vasodilators might indirectly improve perfusion by promoting collateral flow or displacing the embolus distally.

Recent studies have explored “cocktail” regimens for IAT. For example, Yang Fangyu et al. (16) reported a high efficacy rate (94%) using a multi-drug protocol comprising urokinase, papaverine, steroids, and nimodipine. Papaverine not only addresses acute spasm but might also work synergistically with urokinase.

### Adverse events: hemorrhagic risk and complication management

Hemorrhage is the most serious complication of IAT, encompassing intracranial hemorrhage and retinal bleeding. The significantly higher incidence of hemorrhagic events in the IAT group within the EAGLE study highlights this inherent risk (11). However, the occurrence of bleeding is multifactorial, influenced by patient comorbidities (e.g., hypertension, diabetes), anticoagulation status, and procedural technique. The absence of hemorrhagic events in our series may be associated with: (1) strict exclusion of high-risk patients (e.g., coagulation disorders, recent cerebral hemorrhage); (2) short microcatheter dwell time (<5 min), minimizing vessel trauma; and (3) standardized post-procedural antiplatelet management avoiding excessive anticoagulation. Furthermore, comprehensive preoperative imaging (e.g., cranial MRI) helps identify asymptomatic infarcts, potentially avoiding thrombolysis in settings prone to hemorrhagic transformation.

Nonetheless, bleeding risk remains a major barrier to the widespread adoption of IAT. Future refinements involving more meticulous patient selection, intraprocedural imaging guidance, and individualized drug dosing are needed to optimize safety.

### Selection of reperfusion strategy

Regarding the integration of our approach into a broader treatment pathway, the recent European Stroke Organization (ESO) guidelines emphasize the potential of IVT within 4.5 h for acute ischemic stroke, including CRAO (17). In a clinical setting, for a patient presenting within this classic window, IVT should be considered as the first-line emergent treatment if there are no contraindications. Our described intra-arterial approach could then be reserved for cases where IVT is contraindicated, has failed, or for patients presenting beyond the IVT window but within a timeframe deemed potentially salvageable for IAT. This sequential or alternative strategy needs to be prospectively evaluated.

### Study limitations

This study has several limitations. As a single-center retrospective analysis with a small sample size (*n* = 20) and the absence of a control group, its findings require validation in prospective randomized trials. Furthermore, as all enrolled patients received IAT, direct comparison with the natural history or conservative management is not feasible. Second, although we utilized logMAR BCVA for objective assessment, visual outcomes in CRAO are influenced by multiple factors, such as the presence of a cilioretinal artery, initial visual acuity level, and occlusion type (e.g., complete vs. incomplete). These variables were not fully stratified in our analysis. Third, the follow-up period was short (1–2 days post-procedure). All patients were subsequently contacted by telephone for follow-up. Four individuals could not be reached; the remainder reported no further visual deterioration or ocular/adnexal symptoms since discharge. However, telephone-based follow-up lacked the granularity required for objective assessment of long-term visual stability and precluded early detection of delayed complications, including neovascular glaucoma. Additionally, angiographic reperfusion assessment lacks the quantitative perfusion parameters provided by modalities like OCTA or fluorescein angiography.

## Conclusion

In conclusion, low-dose urokinase combined with papaverine administered via ophthalmic artery thrombolysis demonstrated promising preliminary efficacy and a favorable safety profile in carefully selected patients with CRAO. Despite ongoing controversy, IAT remains a treatment strategy worthy of further exploration. Future research should focus on conducting multicenter prospective randomized trials with stratification based on CRAO type to definitively identify the patient population that benefits most; investigating safer thrombolytic protocols.

## Data Availability

The raw data supporting the conclusions of this article will be made available by the authors, without undue reservation.
